# Preoperative Chemotherapy and Its Efficacy in Pediatric Wilms Tumor: A Systematic Review of Randomized Controlled Trials

**DOI:** 10.7759/cureus.117605

**Published:** 2026-10-07

**Authors:** Deema Alqahtani, Saja Alqahtani, Rama Hidayah, Mohammed Alghirash, Redwan M Mulla, Sama Alsaadi, Fakhirah Alhajri, Dima Alluhaidan, Alanoud Barayan, Shouq Naif Aloufi, Shoug Alharbi, Ihab Sulaiman

**Affiliations:** 1 Medicine and Surgery, King Saud University, Riyadh, SAU; 2 Medicine and Surgery, Jazan University, Jazan, SAU; 3 Medicine and Surgery, University of Jeddah, Jeddah, SAU; 4 Medicine and Surgery, King Faisal University, Al-Ahsa, SAU; 5 Pediatrics, Taibah University, Madinah, SAU; 6 Medicine and Surgery, Qassim University, Qassim, SAU; 7 Medicine and Surgery, King Saud bin Abdulaziz University for Health Sciences, Jeddah, SAU; 8 Medicine, Taibah University, Madinah, SAU; 9 Pediatrics, Prince Sultan Military Medical City, Riyadh, SAU; 10 Cardiology, King Abdulaziz Medical City Riyadh, Riyadh, SAU

**Keywords:** event-free survival, neoadjuvant therapy, nephroblastoma, pediatric oncology, preoperative chemotherapy, tumor shrinkage, wilms tumor

## Abstract

Wilms tumor (nephroblastoma) is the most common malignant renal tumor in children. Preoperative (neoadjuvant) chemotherapy has been widely adopted to reduce tumor size, facilitate surgical resection, and decrease the risk of intraoperative tumor rupture. However, the magnitude of its clinical benefits remains a subject of debate. This systematic review aimed to evaluate the effectiveness of preoperative chemotherapy in children with Wilms tumor in terms of tumor shrinkage, complete tumor resection, and event-free survival (EFS). This systematic review was conducted according to the Preferred Reporting Items for Systematic Reviews and Meta-Analyses (PRISMA) guidelines. PubMed, Google Scholar, and ScienceDirect were searched for randomized controlled trials (RCTs) published up to 2024 that evaluated preoperative chemotherapy in pediatric patients with Wilms tumor. Eligible studies compared preoperative chemotherapy with upfront surgery, alternative chemotherapy strategies, or different durations of preoperative treatment. Data extraction and risk-of-bias assessment were performed independently by two reviewers. Due to the limited number of studies and substantial clinical heterogeneity, a narrative synthesis was conducted instead of a meta-analysis. Three RCTs involving 631 pediatric patients were included. Preoperative chemotherapy consistently resulted in significant tumor shrinkage and facilitated surgical management. A reduction in intraoperative complications, particularly tumor rupture, was observed in patients receiving preoperative therapy compared with upfront surgery. Survival outcomes were generally comparable between treatment groups, with no significant improvement in EFS or overall survival (OS) associated with prolonged preoperative treatment. Evidence from one trial demonstrated improved tumor response and reduced perioperative blood loss with trans-arterial chemoembolization combined with surgery. Overall, extending preoperative chemotherapy beyond four weeks did not provide additional clinical benefit. These findings suggest that preoperative chemotherapy in pediatric Wilms tumor improves tumor shrinkage and surgical feasibility while reducing operative complications. Current evidence does not support extending treatment duration beyond standard short-course regimens, as longer courses do not appear to improve survival outcomes and may increase treatment-related toxicity. Further high-quality randomized studies are needed to strengthen the evidence base and clarify long-term outcomes.

## Introduction and background

Wilms tumor, referred to as nephroblastoma, is the most prevalent malignant kidney tumor in childhood, affecting mostly children aged under five years [1]. The worldwide age-standardized incidence is estimated at approximately 7.7-8.3 cases per year per million children aged under 15 years [2].

At the national level, there are no specific population-based studies in Saudi Arabia that accurately document the incidence of Wilms tumor in children. However, national cancer registry data indicate that the overall incidence of pediatric cancer among individuals younger than 20 years is approximately 15.3 cases per 100,000 annually. Wilms tumor is the most common malignant renal tumor in children and may occur as part of several congenital syndromes [3]. It most commonly presents between three and four years of age, although clinical presentation is variable and may include an abdominal mass or swelling, abdominal pain, hematuria, fever, anemia, or hypertension. Diagnosis is typically established through radiologic imaging followed by histopathological confirmation [4,5].

Over the last few decades, treatment outcomes for children with Wilms tumor have significantly improved. In developed countries, five-year survival rates exceed 90% as a result of advances in multimodal therapy combining surgery, chemotherapy, and radiotherapy [6]. Preoperative chemotherapy, administered before surgical tumor removal, has received considerable attention because of its potential to reduce tumor size, facilitate safer resection, and decrease the risk of intraoperative tumor rupture [7].

Leading cooperative groups, including the International Society of Pediatric Oncology (SIOP) and the National Wilms Tumor Study Group (NWTS), have extensively investigated preoperative chemotherapy in children with Wilms tumor. Previous studies have reported benefits in tumor reduction, surgical accessibility, and reduction of intraoperative tumor rupture [8,9]. However, uncertainty remains regarding its clinical benefit, particularly survival outcomes and the optimal duration of treatment.

Randomized trials, including SIOP WT-2001 and the UKW3 trial, suggested that preoperative chemotherapy could downgrade tumors, reduce the risk of stage III disease, and improve surgical accessibility in difficult anatomical situations [10]. However, response and downstaging rates did not appear to improve beyond four weeks, suggesting a potential optimal duration of treatment [11].

Despite these potential benefits, preoperative chemotherapy has limitations. Early exposure to cytotoxic agents may alter tumor histology, affect accurate staging, or mask unfavorable subtypes [11]. Preoperative chemotherapy may cause toxic effects that require additional time for recovery, potentially delaying definitive surgery [12]. Therefore, this systematic review aimed to assess whether preoperative chemotherapy improves tumor shrinkage, facilitates complete tumor resection, and improves event-free survival (EFS) in children with Wilms tumor.

## Review

Methodology

This systematic review followed the Preferred Reporting Items for Systematic Reviews and Meta-Analyses (PRISMA) guidelines to maintain consistency and clarity while selecting and reporting studies [13]. The review protocol was registered in PROSPERO under the ID: CRD420251088464 [14].

Since the work relied on previously published research and did not involve any direct interaction with human participants, ethical approval was not required. The review was completed in 2025.

Search Strategy

A structured search was carried out in PubMed, Google Scholar, and ScienceDirect in August 2025 to locate RCTs that examined the use of preoperative (neoadjuvant) chemotherapy in children diagnosed with Wilms tumor. The screening process was facilitated using Rayyan (Rayyan Systems Inc., Cambridge, MA, USA), a web-based platform that enables independent and blinded study selection [15].

The search strategies used for each database, including the search terms, fields searched, and filters or limits applied, are presented in Table 1.

**Table 1:** Search strategy applied across the electronic databases EFS: event-free survival.

Database	Search string/terms used	Fields searched	Filters/limits applied
PubMed	("Wilms tumor" OR "nephroblastoma") AND ("preoperative chemotherapy" OR "neoadjuvant therapy") AND ("tumor shrinkage" OR "resection" OR "event-free survival" OR "EFS")	Title/abstract	English language; publication date up to 2024; human studies; randomized controlled trials
Google Scholar	("Wilms tumor" OR "nephroblastoma") AND ("preoperative chemotherapy" OR "neoadjuvant therapy") AND ("tumor shrinkage" OR "resection" OR "event-free survival" OR "EFS")	Title/abstract/full text	English language; publication date up to 2024; randomized controlled trials
ScienceDirect	("Wilms tumor" OR "nephroblastoma") AND ("preoperative chemotherapy" OR "neoadjuvant therapy") AND ("tumor shrinkage" OR "resection" OR "event-free survival" OR "EFS")	Title/Abstract/Keywords	English language; publication date up to 2024; randomized controlled trials

The study selection followed the Population, Intervention, Comparison, Outcomes, and Study Design (PICOS) framework [16]. Reference lists of relevant articles were also reviewed to identify any additional studies, as shown in Table 1, which presents the search strategy used for each database.

Eligibility Criteria

Studies were considered eligible for inclusion if they met the predefined eligibility criteria according to the PICOS framework. Eligible studies were those published in the English language and available up to the year 2024. The study population was required to consist of pediatric patients younger than 18 years old with a confirmed diagnosis of Wilms tumor. Studies were included if they evaluated preoperative (neoadjuvant) chemotherapy as the primary intervention and compared it with either upfront surgery or alternative chemotherapy regimens. In addition, studies were required to report at least one of the predefined primary outcomes of interest, including tumor shrinkage, complete tumor resection, or EFS. Only RCTs were considered eligible for inclusion, in accordance with the study selection criteria.

Studies were excluded if they involved adult populations or patients without a confirmed diagnosis of Wilms tumor, did not evaluate preoperative (neoadjuvant) chemotherapy, lacked a clearly defined comparator group, or failed to report any of the predefined clinical outcomes. Furthermore, studies with non-eligible designs were excluded, including case reports, case series, editorials, commentaries, duplicate publications, narrative reviews, systematic reviews, meta-analyses, animal studies, and laboratory or cadaveric research, as illustrated in Table 2.

**Table 2:** Inclusion and exclusion criteria applied during the study selection process

Inclusion criteria	Exclusion criteria
Pediatric patients diagnosed with Wilms tumor	Studies involving adults
Studies evaluating preoperative chemotherapy	Studies not evaluating preoperative chemotherapy
Randomized controlled trials	Observational studies, case reports, case series, reviews, and editorials
Studies comparing preoperative chemotherapy with upfront surgery or another treatment strategy	Studies without a relevant comparison group
Studies reporting relevant clinical or surgical outcomes	Studies that did not report relevant outcomes
Full-text articles available	Conference abstracts without sufficient data
Studies published in English	Articles published in languages other than English

Study Selection

A comprehensive literature search was conducted across three electronic databases, namely, PubMed, Google Scholar, and ScienceDirect. Following the removal of duplicate records, all retrieved titles and abstracts were screened for relevance. Following the initial screening, full-text articles were evaluated for eligibility based on the predefined inclusion and exclusion criteria. The detailed study selection process is presented in Figure 1 (PRISMA flow diagram).

**Figure 1:**
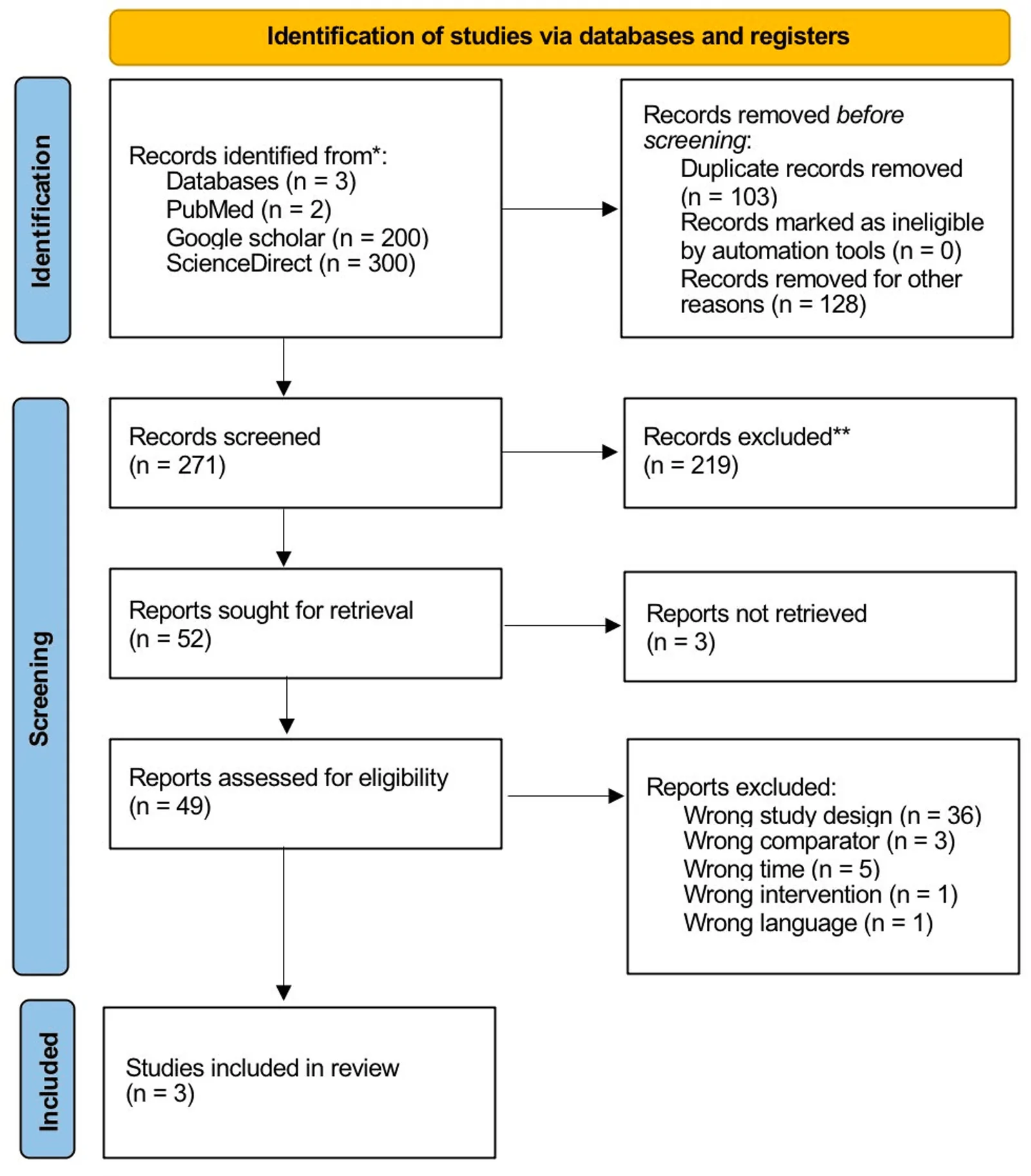
PRISMA 2020 flow diagram of the study selection process summarizing the literature search, screening process, eligibility assessment, and final inclusion of studies in this systematic review *Details of search sources and number of records. ⁠**Number of records excluded during screening. PRISMA: Preferred Reporting Items for Systematic Reviews and Meta-Analyses.

Study Screening

Two independent reviewers screened all studies retrieved through the literature review, starting with title and abstract screening to determine whether each article met the initial eligibility criteria. Any disagreements in their evaluations were discussed, and if they could not reach agreement, a third reviewer was consulted. The full-text articles that appeared eligible after this first phase were reviewed by the same two reviewers working separately, using the same process to resolve any differences.

Data Extraction

To ensure consistency across studies, a standardized data extraction form was used for data extraction. The study characteristics (title, authors, year of publication, and country), study design, sample size, patient demographics, characteristics of the preoperative (neoadjuvant) chemotherapy regimen, comparator groups, and the key clinical outcomes (tumor shrinkage, complete tumor resection, and EFS) were collected from each study. All identified variables were extracted independently by two reviewers, and discrepancies between their findings were discussed and, if necessary, resolved with a third reviewer.

Risk-of-Bias Assessment

Because all included studies were RCTs, the Cochrane Risk of Bias 2 (RoB 2) tool was used to evaluate the methodological quality and risk of bias of each included study. This assessment covered five key domains: the randomization process, deviations from intended interventions, missing outcome data, measurement of outcomes, and selection of reported results. Each study was independently assessed by two reviewers to ensure accuracy and consistency. Any disagreements were resolved through discussion and consensus. The overall risk of bias for each trial was categorized as “low risk,” “some concerns,” or “high risk,” according to the RoB 2 guideline. The detailed risk of bias assessment is presented in Figure 2.

**Figure 2:**
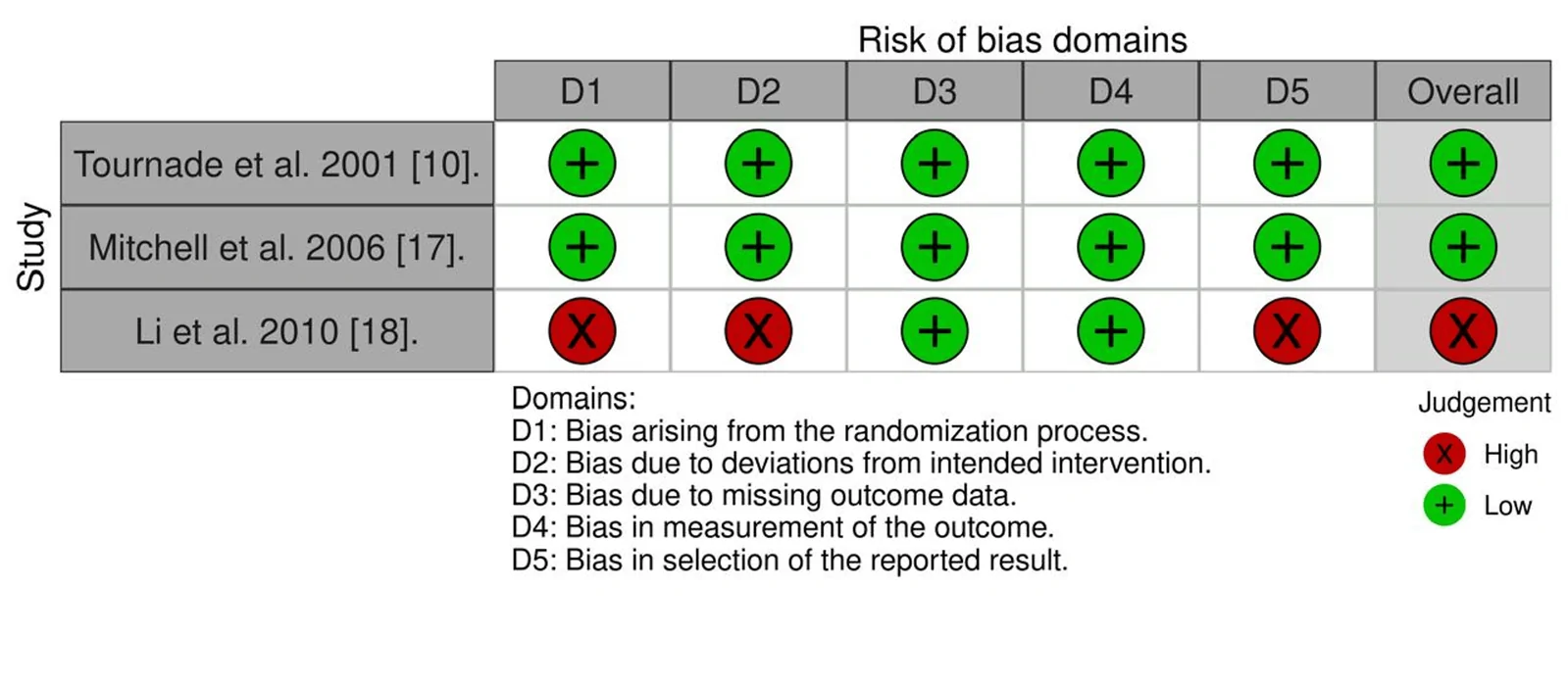
Risk-of-bias assessment of the included randomized controlled trials The risk-of-bias assessment was performed using the Cochrane Risk of Bias 2 (RoB 2) tool, with judgments categorized as low risk, some concerns, or high risk across the assessed domains [10,17,18].

Data Synthesis

A quantitative meta-analysis was planned as part of this systematic review. However, a meta-analysis was not performed because of the limited number of eligible RCTs and the substantial clinical and methodological heterogeneity among the included studies. Differences in interventions, comparator groups, outcome reporting, and follow-up periods prevented meaningful statistical pooling of the data. Therefore, the findings were synthesized and presented using a narrative approach. The findings were descriptively summarized and compared across the included studies according to the intervention and comparator groups and the reported clinical outcomes, including tumor shrinkage, complete tumor resection, EFS, overall survival (OS), recurrence, and treatment-related complications.

Result

Our review included three RCTs evaluating preoperative chemotherapy in pediatric Wilms tumor. The Tournade et al. multicenter SIOP trial (n = 382) compared four-week preoperative chemotherapy (n = 193) with eight-week preoperative chemotherapy (n = 189), with outcomes including tumor shrinkage, stage distribution, EFS, OS, and treatment-related complications over five years [10]. Mitchell et al. (n = 205) compared preoperative chemotherapy (n = 103) with upfront surgery (n = 102), with a median follow-up of 7.2 years and assessment of survival, surgical morbidity, and treatment-related outcomes. Li et al. evaluated trans-arterial chemoembolization (TACE) plus surgery (n = 22) versus surgery alone (n = 22), with a mean follow-up of approximately 3.7 and 3.5 years, respectively, and assessed tumor response, perioperative blood loss, and clinical outcomes. As summarized in Table 3, the included studies differed in their intervention characteristics, comparator strategies, and chemotherapy protocols.

**Table 3:** Characteristics of included randomized controlled trials evaluating preoperative chemotherapy in pediatric Wilms tumor patients QoL: quality of life, OS: overall survival, RCT: randomized controlled trial, TACE: trans-arterial chemoembolization.

Author (year)	Study design	Country	Total (N)	Group 1 (N)	Group 2 (N)	Outcomes measured	Follow-up
Tournade et al. (2001) [10]	RCT	Multicenter (SIOP)	382	193 (4-week pre-op chemo)	189 (8-week pre-op chemo)	Stage I tumor %, tumor shrinkage, complete resection, EFS, OS, surgical complications, recurrence, toxicity	5 years
Mitchell et al. (2006) [17]	RCT	Australia, Ireland, Norway, UK	205	103 (pre-op chemo)	102 (upfront surgery)	Tumor stage distribution, need for radiotherapy/doxorubicin, EFS, OS, surgical morbidity (rupture), toxicity, relapse rates	Median 7.2 years (range: 48 days-12.1 years)
Li et al. (2010) [18]	RCT	China	44	22 (TACE pre-op chemoembolization)	22 (surgery only)	Tumor-free survival, recurrence, mortality, tumor shrinkage, necrosis %, apoptosis/mitotic index, fibrosis, complications, QoL	Mean ~3.7 vs 3.5 years

Table 4 shows the baseline demographic and clinical characteristics of the included trials. Tournade et al. included children aged 6 months-16 years with unilateral, non-metastatic Wilms tumor, predominantly Stage I-III disease; Stage I disease was reported in 64% of patients in the four-week group and 62% in the eight-week group [10]. In Mitchell et al., patients ranged from 6 months to 14 years, with a median age of 2.8 years and a near-equal sex distribution (approximately 52% male and 48% female); most patients had favorable histology [17]. Li et al. included younger patients aged 6 months-8 years, with mean ages of 2.7 ± 1.8 years in the TACE group and 2.8 ± 2.5 years in the surgery-only group, and included 31 males and 13 females [18]. Histological risk categories ranged from low to high risk, with three cases of anaplastic histology reported. Across the included trials, eligibility criteria generally included patients with non-metastatic and resectable disease.

**Table 4:** Baseline demographic and clinical characteristics of pediatric patients included in randomized controlled trials WT: Wilms tumor, NWTS: National Wilms Tumor Study, IVC: inferior vena cava.

Author (year)	Age range	Mean age (years ± SD)	Male (n)	Female (n)	Comorbidities	Histology	Inclusion/exclusion criteria	Tumor stage
Tournade et al. (2001) [10]	6 months-16 years	Not reported	Not reported	Not reported	Not reported	Favorable, standard, unfavorable	Inclusion: age 6 months-16 years, unilateral tumor, radiologic WT, no metastases, response after 4 weeks chemo. Exclusion: age <6 months or >16 years, uncertain diagnosis, post-nephrectomy registration, bilateral/stage IV disease, inability to comply	Stage I-III. Stage I: 64% (4-week) vs 62% (8-week), others; Stage II-III
Mitchell et al. (2006) [17]	6 months-14 years	Median: 2.8 years	~52%	~48%	Not reported	Predominantly favorable; small proportion unfavorable and non-WT tumors	Inclusion: age 6 months-16 years, non-metastatic resectable tumor. Exclusion: metastases, bilateral tumors, IVC involvement, diagnostic uncertainty, large fixed tumors	Not explicitly detailed
Li et al. (2010) [18]	6 months-8 years	TACE: 2.7 ± 1.8; control: 2.8 ± 2.5	31	13	Not reported	Low-, intermediate-, high-risk; 3 anaplastic cases	Inclusion: WT patients (1992-2008), staged (NWTS-3), randomized, consent obtained. Exclusion: not specified	TACE: Stage II-IV; Control: Stage II-IV; no Stage I

Table 5 shows the preoperative strategies and chemotherapy protocols used in the included trials. In Tournade et al. and Mitchell et al., preoperative chemotherapy consisted of vincristine plus actinomycin D, with doxorubicin added for patients with unfavorable histology in Mitchell et al. In Li et al., intra-arterial doxorubicin and vincristine were administered via TACE. The duration of preoperative therapy was four weeks versus eight weeks in Tournade et al., approximately six weeks in Mitchell et al., and a single TACE session in Li et al. The comparator strategies included shorter versus longer preoperative chemotherapy, delayed versus immediate nephrectomy, and TACE plus surgery versus surgery alone. The timing of surgery varied across studies, occurring at week 5 versus week 9 in Tournade et al., immediately or after six weeks of chemotherapy in Mitchell et al., and approximately two weeks after TACE in Li et al. Postoperative chemotherapy was consistently risk-adapted, which ranged from minimal therapy in low-risk disease to prolonged regimens up to 12 months in higher-risk groups, while Li et al. applied a uniform six-cycle approach [10,17,18].

**Table 5:** Preoperative strategies and chemotherapy protocols used in included randomized controlled trials VCR: vincristine, AMD: actinomycin D, FH: favorable histology, RT: radiotherapy, AV: actinomycin D + vincristine, AVE: actinomycin D + vincristine + etoposide, DEVI: doxorubicin + etoposide + vincristine + ifosfamide.

Author (year)	Intervention type	Preoperative regimen	Duration	Comparator	Treatment duration	Timing before surgery	Upfront surgery	Other comparator	Postoperative chemotherapy
Tournade et al. [10]	Pre-op chemotherapy	Vincristine (VCR) + actinomycin D (AMD)	4 vs 8 weeks	4-week vs 8-week pre-op chemo	4 vs 8 weeks	Surgery at week 5 (4-week) vs week 9 (8-week)	No	Short vs long course	Stage-adapted: none (Stage I FH); AV (Stage I standard/anaplastic); AVE ± RT (Stage II-III); DEVI for anaplastic/clear-cell
Mitchell et al. (2006) [17]	Pre-op chemotherapy vs upfront surgery	VCR weekly ×6 + AMD every 3 weeks ± doxorubicin (if unfavorable histology)	~6 weeks pre-op; 6-12 months post-op	Delayed nephrectomy vs immediate nephrectomy	Pre-op: 6 weeks; post-op: 6 or 12 months	Surgery immediately (upfront) or after 6 weeks chemo	Yes (control arm)	Short (6 months) vs long (12 months) adjuvant chemo	Stage- and histology-based (VCR ± AMD ± Doxorubicin; RT for Stage III)
Li et al. (2010) [18]	TACE (pre-op chemoembolization)	Doxorubicin + vincristine intra-arterial+ embolization (gelatin sponge, lipiodol)	Single session	TACE + surgery vs surgery only	Single pre-op session + 6 cycles post-op	Surgery ~2 weeks after TACE	Yes (control arm)	Not applicable	All patients: 6 cycles systemic chemotherapy

Table 6 shows clinical outcomes across the included trials. Tumor shrinkage exceeded 50% after 4 weeks in Tournade et al. and averaged 48.2% in Li et al. Complete resection was achieved via radical nephrectomy, although rates were not uniformly reported. EFS was 84% versus 83% at two years in Tournade et al. and 79.6% in both arms at five years in Mitchell et al., while Li et al. reported two-year tumor-free survival of 83% versus 33%. OS was 92% versus 87% at five years in Tournade et al. and 89% in Mitchell et al. Tumor rupture occurred in 0% versus 14.6% of patients in Mitchell et al. Recurrence rates varied across the included trials. Treatment-related adverse events included veno-occlusive disease (VOD), with fatal cases reported. Further details on recurrence and treatment-related adverse events are presented in the Appendices.

**Table 6:** Clinical outcomes across the studies included. Primary outcomes consistently showed no survival advantage with prolonged preoperative chemotherapy HR: hazard ratio, CI: confidence interval, VOD: veno-occlusive disease, GI: gastrointestinal, N0: node-negative, N/V: nausea and vomiting.

Author (year)	Primary outcomes	Tumor shrinkage	Complete resection	Time points	Event-free survival (EFS)	Overall survival (OS)	Operative complications	Recurrence	Adverse effects
Tournade et al. (2001) [10]	No benefit of prolonged pre-op chemo; 4-week regimen recommended	>50% shrinkage after 4 weeks; additional >50% reduction with further 4 weeks	Radical nephrectomy performed (rate not specified)	Not clearly specified	2-year: 84% (4-week) vs 83% (8-week)	5-year: 92% (4-week) vs 87% (8-week)	Tumor rupture: 1% (4-week) vs 3% (8-week)	~5% overall; Stage II N0: 6.6%	Hepatic toxicity, thrombocytopenia, leukopenia, anemia, GI, neurologic; VOD; 1 infection-related death
Mitchell et al. (2006) [17].	Improved stage distribution with pre-op chemo; reduced treatment burden without survival compromise	Not reported	Not reported	Stage: post-surgery; survival from randomization; median follow-up 7.2 years	5-year: 79.6% both groups; HR 1.25 (95% CI 0.68-2.30)	5-year: 89.0%; HR 0.84 (95% CI 0.38-1.88)	Tumor rupture: 0% (pre-op) vs 14.6% (15/103 upfront); 1 death (VOD)	18.3% (17/93) upfront vs 25.3% (23/91) delayed	Hepatic VOD (2 fatal cases), treatment-related toxicity
Li et al. (2010) [18].	Tumor necrosis, apoptosis, clinical outcomes, perioperative blood loss	Mean shrinkage: 48.2% (TACE)	Not reported	2-year survival; recurrence/death within 1 year; follow-up ~44 vs 42 months	2-year tumor-free survival: 83% (15/18) vs 33% (5/15), p < 0.001	Not reported	Blood loss: 142.2 mL (TACE) vs 258.2 mL (control); no major complications	5.6% (1/18) vs 26.7% (4/15), p < 0.01	Pain (18), fever (16), leukopenia (11), N/V (5), stomatitis (1), alopecia (2), diarrhea (2)

Discussion

Principal Findings

Our findings confirm that preoperative chemotherapy for pediatric Wilms tumor achieves significant tumor shrinkage and effective downstaging, thereby reducing surgical morbidity and intraoperative complications. Furthermore, extending neoadjuvant chemotherapy from the standard four-week regimen to an eight-week regimen did not provide additional oncological or surgical benefit. These findings suggest that shorter preoperative treatment protocols are sufficient to optimize surgical outcomes while minimizing unnecessary chemotherapy exposure and treatment-related toxicity.

Comparison With Previous Evidence

Our findings are consistent with previous randomized trials. Tournade et al. demonstrated that preoperative chemotherapy reduced tumor size and lowered the incidence of intraoperative tumor rupture without improving survival outcomes [10]. Importantly, extending treatment from four to eight weeks did not provide additional clinical benefit, supporting the use of shorter treatment courses. Similarly, Mitchell et al. reported that preoperative chemotherapy eliminated intraoperative tumor rupture while maintaining comparable EFS and OS between treatment groups [17. In contrast, Li et al. evaluated TACE combined with surgery rather than conventional systemic chemotherapy [18]. Their findings demonstrated improved tumor response, reduced perioperative blood loss, and higher short-term tumor-free survival compared with surgery alone, suggesting that alternative neoadjuvant approaches may also improve surgical outcomes in selected patients.

The findings from Tournade et al. and Mitchell et al. also indicate that the reduction in surgical complications and treatment burden associated with preoperative chemotherapy did not translate into measurable improvements in long-term survival [10,17]. In Li et al., the improved tumor response and reduced perioperative blood loss with TACE further suggest potential benefits of alternative neoadjuvant approaches in selected patients [18].

Clinical Implications

Overall, the available randomized evidence supports current treatment strategies favoring standard short-course preoperative chemotherapy. Prolonging treatment beyond four weeks appears unlikely to provide additional oncological benefit while potentially increasing cumulative chemotherapy exposure, treatment-related toxicity, and delays to definitive surgery. These findings may assist clinicians in balancing treatment efficacy with patient safety when selecting the duration of preoperative therapy.

The observed tumor shrinkage and improved surgical feasibility may reduce operative complications even when improvements in EFS or OS are not observed. Alternative neoadjuvant approaches, such as TACE, may also improve tumor response and surgical outcomes in selected patients.

Strengths and Limitations

This review has several strengths. It included only RCTs, followed a predefined protocol registered in PROSPERO, adhered to the PRISMA 2020 guidelines, and used duplicate study selection, data extraction, and risk-of-bias assessment to minimize methodological bias. Restricting the review to randomized evidence also improved the overall quality and reliability of the findings.

However, this review has several limitations. First, only three RCTs met the eligibility criteria, limiting the strength and generalizability of the available evidence. Second, considerable clinical and methodological heterogeneity existed among the included studies regarding chemotherapy protocols, comparator groups, outcome definitions, and follow-up duration, precluding quantitative meta-analysis. Third, only English-language studies were included, which may have introduced language bias. Finally, the limited number of studies prevented subgroup analyses according to tumor stage, histological subtype, or chemotherapy regimen.

## Conclusions

Preoperative chemotherapy in children with Wilms tumor is associated with substantial tumor shrinkage and reduced surgical complications, particularly tumor rupture, without improving EFS or OS. Extending preoperative chemotherapy beyond the standard short-course regimen provides no additional demonstrated benefit. Further randomized studies are needed to clarify the optimal duration of preoperative chemotherapy.

## Appendices

**Table 7:** Detailed treatment protocols, toxicity profiles, and follow-up metrics The studies used different chemotherapy regimens and treatment durations, with varying toxicity profiles and follow-up periods [10,17,18].

Author (year)	Detailed chemotherapy protocols	Toxicity details	Follow-up details
Tournade et al. (2001) [10]	AV, AVE ± RT, DEVI regimens based on stage and histology	Hepatic toxicity, thrombocytopenia, leukopenia, anemia, GI, neurologic, VOD; 1 infection-related death	5-year follow-up
Mitchell et al. (2006) [17]	Risk-adapted regimens: VCR ± AMD ± doxorubicin; 6 vs 12 months duration; RT for Stage III	Hepatic VOD (2 fatal cases), treatment-related toxicity	Median 7.2 years (range up to 12.1 years); continuous monitoring
Li et al. (2010) [18]	Intra-arterial chemo (doxorubicin + vincristine) + embolization; 6 cycles post-op chemo	Pain (18), fever (16), leukopenia (11), N/V (5), stomatitis (1), alopecia (2), diarrhea (2)	Mean follow-up: 44.3 vs 41.7 months; recurrence/death within 1 year
